# A Systematic Review of the Perinatal Mental Health Outcomes of Women With Neurodevelopmental Disorders

**DOI:** 10.1192/bjo.2024.200

**Published:** 2024-08-01

**Authors:** Isobel Mackintosh, Sasha Reed, Surangi Jayakody

**Affiliations:** University of Warwick, Coventry, United Kingdom

## Abstract

**Aims:**

Neurodivergent women have different experiences during pregnancy, childbirth, and parenthood than neurotypical women. However, little is known about the perinatal mental health outcomes and parenting experiences in women with Neurodevelopmental Disorders (ND). The systematic review aimed to summarise the literature on perinatal mental health outcomes and parenting experiences among women with ND.

**Methods:**

MEDLINE, Embase and PsycINFO databases were searched in October 2023 using the keywords related to pregnancy outcomes, perinatal period, mental health, neurodivergent, and neurodevelopmental disorders. Papers were also identified through citation and/or hand searching. Title, abstracts, and full-text articles were independently screened by two authors, and data were extracted using a custom data extraction spreadsheet. The Joanna Briggs Institute and the Mixed Methods appraisal tools were used for the critical appraisal. The heterogeneity across the included studies ruled out the use of meta-analysis. Therefore, results were summarised using a narrative synthesis.

**Results:**

Fourteen studies were included in the final review; four cohort, four case-control, three cross-sectional and three qualitative studies across 940,354 participants. The studies investigated women with Autism, Asperger's syndrome and Attention-Deficit Hyperactivity Disorder (ADHD), who were either clinically diagnosed or scored appropriately on diagnostic questionnaires. Perinatal mental health outcomes covered anxiety and depression. These were measured using questionnaires such as the Edinburgh Postnatal Depression Scale, participant interviews and clinical diagnosis from qualified healthcare professionals. All fourteen studies found a correlation between Neurodevelopmental Disorders and perinatal anxiety and/or depression symptoms. Seven studies found that neurodivergent women had adverse pregnancy and early parenting experiences. Results suggested this correlation may be mediated by factors such as unsatisfactory healthcare, lack of maternal-infant bond, increased sensory overload, issues with emotional attachment, difficulty reading the facial expression of the baby and problems with breastfeeding. Overall, women with ND were more likely to feel anxious and overwhelmed during the perinatal period, a potential risk factor for perinatal mental illness.

**Conclusion:**

Women with ND are at a higher risk of developing perinatal mental illness and adverse early parenting experiences. Abnormal physical and sensory challenges during pregnancy as well as difficulty with emotional connection and infant bonding during postpartum all contribute to the increased risk of perinatal mental illness. Adaptations to appointments and specialised perinatal care are required for women with ND yet are often not provided. To reduce the risk of perinatal mental illness in women with ND, improvements must be made to the delivery of perinatal care and the knowledge of those providing the care.

